# From work-related trauma to suicidal ideation: a serial mediation model of posttraumatic stress and depression in rescue workers

**DOI:** 10.3389/fpsyt.2026.1805837

**Published:** 2026-04-10

**Authors:** Leila M. Soravia, Gianluca A. Florineth, Thomas Müller, Sebastian Walther, Mario Pfammatter, Niklaus Denier, Tobias Bracht, Kristina Adorjan

**Affiliations:** 1 Translational Research Center, University Hospital of Psychiatry, University of Bern, Bern, Switzerland; 2 Competence Center for Interventional Psychiatry and Augmented Psychotherapy, University Hospital of Psychiatry, University of Bern, Bern, Switzerland; 3 Graduate School for Health Sciences, University of Bern, Bern, Switzerland; 4 Private Clinic Meiringen, Meiringen, Switzerland; 5 Department of Psychiatry, Psychosomatics, and Psychotherapy, Center of Mental Health, University Hospital of Würzburg, Würzburg, Germany

**Keywords:** depressive symptoms, post-traumatic stress symptoms, rescue workers, suicidality, trauma

## Abstract

**Objectives:**

Rescue workers face frequent occupational trauma, increasing their risk for posttraumatic stress symptoms (PTSS), depression, and suicidal ideation. However, pathways linking trauma to suicidality remain poorly understood. This study investigated these mechanisms by testing a serial mediation model.

**Methods:**

From a larger survey of Swiss rescue workers, participants reporting suicidal ideation (n = 44) were matched by age, sex, and profession with a control group without suicidal ideation (n = 44). Symptomatology was assessed using validated questionnaires such as the Posttraumatic Stress Scale-10 (PTSS-10) for posttraumatic stress and the Brief Symptom Inventory (BSI) for depressive symptoms. Structural Equation Modeling (SEM) was employed to test a serial two-mediator model: Trauma Exposure - PTSS - Depressive Symptoms - Suicidal Ideation.

**Results:**

Participants with suicidal ideation had significantly higher levels of trauma, PTSS, and depressive symptoms. SEM confirmed an excellent model fit (χ² = 1.925, CFI = 1.000, RMSEA <.001) and a full mediation effect: trauma exposure was associated with PTSS, which in turn related to depressive symptoms, which were subsequently linked to suicidal ideation. The specific serial indirect pathway was significant (B = 0.143, p = .011), while the direct path from trauma to suicidal ideation was non-significant. The model explained 69.4% of the variance in suicidal ideation.

**Conclusion:**

The findings suggest a developmental pathway in which trauma exposure is associated with suicidal ideation through the sequential roles of PTSS and depressive symptoms. Consequently, suicide prevention for rescue workers should prioritize the management of post-traumatic and depressive symptoms to potentially disrupt this symptomatic progression.

## 1 Introduction

Rescue workers experience elevated occupational stress and are frequently exposed to traumatic events, placing them at substantially increased risk of developing psychiatric disorders (1–3). The most notable consequences include posttraumatic stress symptoms (PTSS) and posttraumatic stress disorder (PTSD), with prevalence estimates for PTSD or suspected PTSD ranging from approximately 10% to over 20% depending on occupation, exposure type, and context (4–6). Beyond PTSD, these individuals are at increased risk for various psychological strains, such as depression, anxiety, burnout, and suicidal ideation (7, 8). A recent meta-analysis by Martinez (9) reports that over 90% of individuals with PTSD present with at least one additional mental disorder, most frequently alcohol dependence (10), depression, or anxiety disorders (11). This risk is heightened by cumulative trauma exposure, frequent involvement in distressing incidents, and specific events such as handling fatalities or being assaulted (1, 4). A cross-sectional study by Soravia et al. (5) highlights that the prevalence of suspected PTSD (based on screening instruments) varies significantly across professions, ranging from 8% in firefighters to 22% in psychiatric staff, and is strongly linked to psychological distress and suicidal thoughts (5). Key risk factors identified included dysfunctional coping strategies, low perceived self-efficacy, prior trauma exposure, and years of service.

As the mental health burden in these professions frequently manifests as suicidality, studies in Australia (12) and the U.S (13–15). suggest that first responders in emergency services report significantly higher rates of suicidal ideation and planning than the general population. Police officers face a 54% higher suicide risk compared to other professionals (14, 15). While German findings support this trend, citing high occupational stress and psychiatric comorbidities as key drivers (16), the broader literature remains divided. Other studies report suicide rates that are comparable to, or even lower than, those of the general population (17, 18). We find a similar picture in nurses. An analysis of U.S. data (19) revealed that nurses had an 18% higher likelihood of dying by suicide than the general population, with female nurses facing double the risk of the general population. Stigma often discourages this group from seeking help (20).

Trauma- and stressor-related disorders are robustly linked with increased risk for suicidal behavior. Meta-analyses consistently demonstrate that individuals with PTSD have approximately twice the risk of suicide compared to the general population, alongside elevated risk for attempts and ideation. Large-scale studies confirm that the lifetime prevalence of suicide attempts among individuals with PTSD is estimated to be around 10% (21–23). However, the pathway from trauma to suicide is complex and often involves comorbidities. For example, a Danish cohort study found that acute stress reaction increased the risk of completed suicide tenfold, an effect exacerbated by depression or substance abuse (21). Furthermore, in a 12-year prospective study, while PTSD and anxiety were associated with suicide attempts, Major Depressive Disorder (MDD) remained as the significant independent predictor in multivariate analyses (24). This highlights the prominent and potentially synergistic role of comorbid mood disorders in the trajectory from trauma to suicide.

In summary, PTSD, depression, anxiety, burnout, and suicidal ideation are the principal psychological consequences of trauma exposure in rescue workers. While risk factors vary by profession and individual characteristics, it remains unclear exactly how these psychopathological factors interact or depend on each other to precipitate suicidal ideation.

This study examines the association between post-traumatic stress symptoms, depressive symptoms, and suicidal ideation among rescue workers. Specifically, we investigate the mediating role of these symptoms in the development of suicidality. We hypothesize that both post-traumatic stress symptoms and depressive symptoms are significantly associated with an increased risk of suicidal ideation in rescue and emergency personnel. We further assume that rescue workers reporting suicidal ideation will exhibit a distinct risk profile characterized by higher rates of suspected PTSD, elevated depressive symptoms, greater cumulative trauma exposure, increased sleep disturbance, higher psychological distress, and more dysfunctional coping behaviors compared to those without suicidal ideation.

## 2 Materials and methods

### 2.1 Participants

Eighty-eight participants were selected for this secondary analysis on post-traumatic stress symptoms, depressive symptoms and suicidality among rescue workers. All participants were part of a large anonymous online study on traumatization among rescue workers in Switzerland (5). The participation in the anonymous online study was voluntary and promoted by the department heads at the places of work corresponding to the studied professions. The study was conducted in accordance with the Declaration of Helsinki. According to the Swiss Federal Act on Research involving Human Beings, the study was exempt from formal ethical review requirements as it involved the collection of anonymous data. Participation was entirely voluntary, and all participants provided consent for the use of their data by completing the survey. The study was registered at ClinicalTrials.gov Nr. NCT03842553. The selected subsample consisted of 44 participants who reported suicidal ideation in the last 12 months and 44 participants without suicidal ideation in the last 12 months matched regarding sex, age, profession and years in job. The final sample consisted of 34 (29.4% female) police officers from the cantonal police forces, 12 (0% female) firefighters from the professional fire service, 12 (16.7% female) ambulance personnel, 6 (33.3% female) emergency staff of the University Hospital of Bern, and 24 (33.3% female) qualified staff of two acute care wards of the University Hospital of Psychiatry in Bern. Of all participants, 56.8% were married or in a stable relationship, 35.2% were single, 6.8% were divorced and 1.1% widowed. The mean age of the study subjects was 40.1 years (SD, 8.7; range, 22-57). Participants reported an average job experience of 15.1 years (SD, 8. 5).

### 2.2 Materials

Participants completed a 20-minute online questionnaire covering socio-demographic characteristics, suicidal ideation, overall psychological strain—including depressive symptoms and sleep disturbances—posttraumatic stress symptoms, perceived self-efficacy, and well-being. Job-specific variables assessed included the number of traumatic events experienced before and during their professional career, years of work experience, coping strategies for managing stressful and traumatic events, and the perceived impact of job-related stressors.

Psychological distress and psychiatric disorders were assessed using the German version of the Brief Symptom Inventory (BSI (25, 26);), a 53-item self-report questionnaire designed to measure symptoms of general psychopathology. For this study, the focus was on three global indices of psychological distress: the Global Severity Index (GSI), the Positive Symptom Distress Index (PSDI), and the Positive Symptom Total (PST).

Depressive symptoms were evaluated using the depressive symptom subscale of the BSI.

Suicidal ideation was assessed by asking participants if they had experienced suicidal thoughts within the 12 months prior to completing the questionnaire and whether they attributed these thoughts to work-related factors.

Sleep disorders were measured using a composite scale derived from items related to sleep difficulties in the BSI (difficulty falling asleep), the PTSS (sleep disturbances), and the GHQ (reduced sleep due to worries).

Posttraumatic stress symptoms were assessed with the German Version of the Post Traumatic Symptom Scale (PTSS-10) (27, 28), a self-rating scale that measures the most common posttraumatic symptoms (sleep disorders, nightmares, fear of reminders, etc.) (29, 30). Attained values above 12.5 were associated with a suspected diagnosis of PTSD (31). Participants further rated whether they had experienced traumatic situations before and during work.

Mental wellbeing was assessed using the German version of the shortest 12-items General Health Questionnaire 12 (GHQ-12 (32, 33);, which was developed to measure current mental wellbeing and is widely used to detect psychological strain in the general population.

Perceived self-efficacy was measured using the General Perceived Self-Efficacy Scale (34), a 10-item self-report instrument for personality diagnostics based on Banduras’s concept of perceived self-efficacy that measures the general optimistic competence expectation (35).

### 2.3 Statistical analyses

The 44 participants who reported suicidal ideation within the 12 months prior to the survey (SI) were matched with 44 participants without suicidal ideation (NoSI) based on sex, age, occupation, and approximate years of employment.

All statistical analyses were conducted using R (version 4.2.3) with the *lavaan* package for Structural Equation Modeling (SEM). As there was no missing data, all analyses included the full sample. The Mean- and Variance-adjusted Weighted Least Squares (WLSMV) estimator was used to accommodate the binary nature of the suicidal ideation variable and to provide robust standard errors given the sample size. Multicollinearity was ruled out by low Variance Inflation Factor (VIF) scores across all regression equations.

#### 2.3.1 Serial mediation model

Our primary hypothesis tested a serial two-mediator model in which trauma exposure predicts posttraumatic stress symptoms, which in turn predict depressive symptoms, and ultimately suicidality. We first estimated a saturated model (df = 0) that included all theoretically plausible direct and indirect paths. For parsimony, two non-significant and non-serial pathways were constrained to zero. The resulting more parsimonious model (df = 2), which focused on the hypothesized serial pathway, was retained for final interpretation.

#### 2.3.2 Model fit and reporting

Model fit was evaluated using robust fit indices, including the scaled chi-square statistic, Comparative Fit Index (CFI), Tucker–Lewis Index (TLI), Root Mean Square Error of Approximation (RMSEA), and Standardized Root Mean Square Residual (SRMR). Model adequacy was judged against conventional criteria, with CFI and TLI ≥ 0.95, RMSEA ≤ 0.06, and SRMR ≤ 0.08 indicating acceptable fit. Both unstandardized (B) and standardized (β) path coefficients are reported. Indirect effect significance was assessed using the delta method.

## 3 Results

### 3.1 Participants

The participants in the two groups (SI vs. NoSI) did not differ in any socio-demographic variables. However, participants with suicidal ideation reported significantly more clinical symptoms, including depressive symptoms, posttraumatic stress symptoms, sleep disorders, greater overall symptom severity compared to those without suicidal ideation, and less perceived self-efficacy (see **Table 1**).

**Table 1:** Demographic and clinical characteristics.

Variable	Total	Group SI	Group NoSI	T/χ ^2^	P
Group size	n = 88	n = 44	n = 44		
Mean age (SD)	40.2 (8.6)	40.3 (8.5)	40.1 (8.7)	0.09	.931
female (%)	22 (25%)	11 (25%)	11 (25%)	0.00	1.00
Mean work experience in years (SD)	15.2 (8.3)	15.3 (9.0)	15.1 (7.7)	0.14	.892
Civil status (%) - married/partner - divorced - single - widowed	51 (58%) 6 (6.8%) 30 (34.1%) 1 (1.1%)	24 (54.5%) 4 (9.1%) 15 (34.1%) 1 (2.3%)	27 (61.4%) 2 (4.5%) 15 (34.1%) 0 (0%)	1.84	.606
Mean BSI GSI (SD)	0.5 (.48)	0.7 (.52)	0.2 (.27)	5.66	<.001
Mean BSI Depr. (SD)	2.9 (3.73)	5.1 (4.00)	0.7 (1.39)	6.98	<.001
Mean Sleep D. (SD)	3.0 (2.33)	3.9 (2.49)	2.1 (1.79)	3.83	<.001
Mean PTSS (SD)	7.6 (5.57)	10.4 (5.42)	4.8 (4.17)	5.42	<.001
Suspected PTSD	23 (26.1%)	19 (43.2%)	4 (9.1%)	13.24	<.001
Mean GHQ (SD)	11.2 (6.14)	14.8 (6.44)	7.6 (2.97)	6.65	<.001
Mean PSES (SD)	30.4 (3.44)	29.3 (3. 379)	31.6 (3.18)	-3.10	.001
Trauma history	30 (34.1%)	16 (36.4%)	14 (31.8%)	.20	.653
One or more trauma(s) during work	80 (90.9%)	43 (97.7%)	37 (84.1%)	4.95	.026

SI, group with suicidal ideation; NoSI, Group without suicidal ideation; PTSS, Posttraumatic Symptom Scale; BSI GSI, Brief Symptom Inventory, Subscale Global Severity Index; BSI Depr, Brief Symptom Inventory, Subscale Depressive Symptoms; Sleep D., Sleep Disorders; GHQ, General Health Questionnaire 12; PSES, General Perceived Self-Efficacy Scale.

### 3.2 Correlation analysis

All four primary study variables were significantly and positively intercorrelated, confirming the basic relationships needed for the mediation analysis. Suicidal Ideation showed strong positive correlations with Depressive Symptoms (r = 0.60, p < 0.001) and Posttraumatic Stress Symptoms (r = 0.52, p < 0.001), while\ Trauma Exposure was moderately correlated with Suicidal Ideation (r = 0.25, p = 0.021) (see supplements **Supplementary Table S1**).

### 3.3 Serial mediation model fit and causal paths

**Figure 1:**
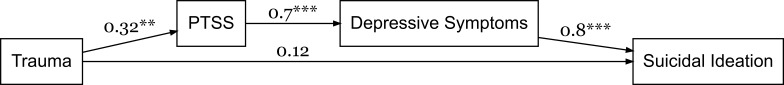
Serial mediation model illustrating the serial indirect effect of trauma exposure on suicidal ideation through posttraumatic stress symptoms (PTSS) and depressive symptoms (BSCL). Standardized path coefficients are displayed for all significant paths. **p<.01; ***p<.001.

Following the evaluation of the fully saturated model (see Supplemental **Supplementary Figure S1**), the hypothesized serial two-mediator model (Trauma Exposure → Posttraumatic Stress Symptoms → Depressive Symptoms → Suicidal Ideation) was tested as a parsimonious model (df = 2) (**Figure 1**). The model demonstrated excellent fit to the data: Scaled χ² (2) = 1.925, p = 0.382, CFI = 1.000, TLI = 1.002, RMSEA = 0.000, and SRMR = 0.007. The non-significant χ² test indicates that the parsimonious model adequately represents the data. However, it is important to note that the mathematically perfect CFI and TLI values are largely an artifact of the model’s low degrees of freedom (df = 2) and its near-saturated nature. Furthermore, the RMSEA was 0.000. However, the confidence interval was wide (90% CI,.000 to.210), reflecting the limited statistical power of the χ² test given the sample size.

Analysis of the individual paths within the model confirmed all three links in the hypothesized serial chain were significant. Specifically, the path from Trauma Exposure to Posttraumatic Stress Symptoms showed a significant positive effect (B = 1.356, β = 0.318, p = 0.006). In turn, Posttraumatic Stress Symptoms had a significant positive effect on Depressive Symptoms (B = 0.471, β = 0.700, p < 0.001), and Depressive Symptoms had a significant positive effect on Suicidal Ideation (B = 0.224, β = 0.797, p < 0.001). The direct effect from Trauma Exposure to Suicidal Ideation was non-significant (B = 0.099, β = 0.123, p = 0.208).

The serial indirect effect of trauma exposure on suicidal ideation through the sequential effects of PTSS and Depressive Symptoms was statistically significant (B = 0.143, β = 0.177, p = 0.011). The robustness of this pathway was confirmed via confidence intervals, which did not cross zero (unstandardized 95% CI, [.033 to.253]). These findings support the hypothesis that the relationship between trauma history and suicidal ideation is fully mediated by the serial sequence of PTSS and depressive symptoms. Overall, the model accounted for a substantial amount of variance in suicidal ideation (R² = 0.694).

To ensure the observed mediation effect was not an artifact of item overlap between the mediator scales (e.g., sleep disturbances in both the PTSS-10 and BSI), we conducted a *post-hoc* sensitivity analysis. After removing potentially overlapping items from both composite scores and re-estimating the model, the serial indirect pathway remained statistically significant (p = 0.018).

## 4 Discussion

The aim of this study was to investigate the association between trauma exposure and suicidal ideation in rescue and emergency personnel. Our findings suggest that traumatic events increase the risk of suicidal ideation only when they trigger a specific sequence of psychological symptoms. A serial mediation model confirmed that the relationship between trauma exposure and suicidal ideation is fully mediated by a sequential chain rather than a direct link. Specifically, trauma exposure is associated with an increase in PTSS, which in turn relates to a rise in depressive symptoms - the immediate and significant predictor of suicidal ideation. Crucially, the direct effect of trauma on suicidality was non-significant, indicating that in this population, trauma only contributes to suicidal ideation when it progresses through post-traumatic stress and evolves into depressive symptoms. Overall, this model demonstrated a very good fit to the data and explained a substantial 69.4% of the variance in suicidal ideation. Furthermore, participants with suicidal ideation were matched to a control group based on age, sex, years of work experience, profession, and civil status. Consequently, as there were no significant differences between the groups regarding these factors, socio-demographic variables can be excluded as primary predictors of suicidal ideation in this sample. However, while the serial mediation model is theoretically consistent with a developmental pathway, the cross-sectional nature of our data precludes definitive causal inferences. Rather than a causal cascade, our findings suggest a strong association where depressive symptoms play a central role in the relationship between trauma exposure and suicidal ideation. Although the results indicate that the association between trauma and suicidal ideation is significantly mediated by depressive symptoms, it is important to note that this does not exclude the existence of other, unmeasured pathways. Suicidality in rescue workers is a multifaceted phenomenon, and further longitudinal research is needed to account for additional contributing factors. Nevertheless, these results underscore the importance of targeted psychological interventions that address PTSS early to potentially disrupt the progression toward depressive symptoms and subsequent suicidal ideation.

Furthermore, as expected, our findings regarding clinical comparisons revealed substantial disparities between the two groups. The SI group exhibited significantly higher severity across all measured clinical domains compared to the NoSI group. Specifically, the SI group reported markedly higher depressive symptoms, more severe sleep disorders, and elevated posttraumatic stress symptoms (PTSS). Furthermore, the prevalence of suspected PTSD was significantly higher in the SI group (43.2%) compared to the NoSI group (9.1%). In terms of resilience factors, the SI group reported significantly lower general perceived self-efficacy. Notably, while trauma history prior to work did not differ between groups, the prevalence of trauma experienced during work was significantly higher in the SI group than the NoSI group.

These findings suggest that the path to suicidal ideation among this workforce is not a random occurrence, nor is it driven by socio-demographic background. Instead, it follows a specific “cascading” pathology. The most critical finding is the presence of full mediation, which offers a clinically optimistic perspective. The fact that the direct link between trauma and suicidal ideation was non-significant suggests that trauma is not a deterministic predictor for the inevitable development of suicidal thoughts. Rather, trauma acts merely as the trigger, requiring the development of posttraumatic stress symptoms, which must then evolve into depressive symptoms before suicidal ideation occurs. This implies that clinical interventions targeting either the posttraumatic stress or depressive symptoms (or symptomatology) stages can successfully break the link to suicide ideation, even after trauma has already occurred.

Within this model, while posttraumatic stress is a major contributing factor, depressive symptoms emerge as the proximal gatekeeper or immediate precursor to suicidal ideation. The high correlation and strong beta weight confirm that depression is the primary fuel for suicidal thoughts in this population. It appears that prolonged posttraumatic stress erodes the individual, leading to the hopelessness characteristic of depression, which may result in suicidal ideation. These findings align with Schmidtke et al. (16), who attribute increased suicidality to high occupational stress and psychiatric comorbidities. While the DSM-5 primarily contextualizes suicidal behavior within depression and borderline personality disorder, recent comprehensive reviews identify it as a transdiagnostic phenomenon associated with over 70 diagnoses (22, 36, 37). This transdiagnostic perspective is particularly relevant to our findings, as it validates the role of PTSS - specifically arising from work-related trauma - as a critical, upstream component of the suicidal trajectory. It supports the view that severe stress responses provide the initial destabilization that eventually manifests as suicidal ideation, rather than ideation being solely a product of depressive pathology.

Regarding the source of this trauma, it is notable that trauma experienced during work significantly differentiated between the groups, whereas pre-existing trauma did not. This distinction suggests that the current occupational environment plays a far more critical role in the participants’ current mental health crisis than their distant pasts. Consequently, this points to the necessity of workplace-specific interventions rather than general screenings for childhood or historical issues. This is partly in contrast to studies reporting that previous traumatic experiences are predictive for the development of later PTSS or PTSD (e.g (5, 38). Our findings suggest, however, that while such experiences relate to trauma symptoms, they may not necessarily trigger the sequential progression toward depressive symptoms and suicidal ideation.

Finally, the erosion of perceived self-efficacy observed in the suicidal ideation group paints a picture of “learned helplessness” within the context of this mediation model. This aligns with findings by Bargai et al. (39), who established that learned helplessness serves as a crucial mediator between trauma exposure and the development of both PTSD and depression. They define this state as a psychological trait resulting from repeated exposure to uncontrollable aversive events, leading to a substantial decrease in the ability to associate one’s actions with positive outcomes. In our sample, individuals’ belief in their ability to cope appears to decline as symptoms progress from trauma to PTSS to depressive symptoms. Bargai et al. (r2007) noted that this state reduces the range of adaptive responses to external demands. Thus, in the current context, the combination of high symptom severity and a low perceived ability to handle them might create a fertile ground for suicidal ideation.

The present study is subject to several limitations that must be considered when interpreting the results. First, as this investigation represents an additional analysis of a subsample derived from the larger study by Soravia et al. (5), several limitations inherent to the original design apply here as well. The study utilizes a cross-sectional design, which precludes the establishment of definitive causal inferences. While the hypothesized serial mediation model (Trauma-PTSS-Depression-Suicidal Ideation) is theoretically grounded, the directionality of these associations cannot be confirmed without longitudinal data. Additionally, while the sample size of N = 88 is relatively small for structural equation modeling, this group represents a specific clinical subgroup identified from a large-scale survey of nearly 1,000 rescue workers, and we utilized a matched-control design to reduce confounding variance and enhance the stability of our comparisons. Furthermore, while we controlled for professional background through a matched-control design, the small sample size precluded the use of multigroup analyses to explore how these pathways might differ across specific emergency occupations; future research with larger cohorts should investigate whether professional roles moderate the relationships between trauma, depressive symptoms, and suicidality.

A specific limitation concerns the assessment of the primary outcome variable and clinical diagnostics. Suicidal ideation was not assessed using a validated multi-item instrument (such as the Columbia-Suicide Severity Rating Scale), but via a single binary item. Statistically, dichotomizing this construct restricts variance and reduces the model’s overall sensitivity. Clinically, a binary metric cannot differentiate between the severity, frequency, or nature of suicidal thoughts (e.g., passive ideation vs. active planning). Consequently, clinical conclusions regarding specific suicide risk should be drawn with caution, and future research should utilize validated, continuous psychometric scales. Furthermore, the study relied exclusively on self-report questionnaires rather than standardized clinical interviews or screenings. Consequently, no formal diagnoses according to ICD-10 or DSM criteria (e.g., for Major Depressive Disorder) could be established. Regarding the temporal sequence, it remains unclear whether suicidal ideation was pre-existing or chronic before the reported work-related traumas occurred. Furthermore, consistent with the limitations noted in the main publication (5), the findings rely on retrospective self-report measures. These are susceptible to recall bias and social desirability, particularly regarding sensitive topics like mental health symptoms and trauma history. The voluntary nature of the online survey, entitled “Traumatization in Emergency Service” may have also introduced a self-selection bias, potentially attracting participants who were already sensitized to the topic or currently symptomatic. Moreover, several potential confounding factors—such as substance use, sleep quality, social support, or specific personality traits—were not accounted for, although they are known to significantly influence the relationship between trauma and suicidality.

Additionally, the lack of differentiation between specific types of trauma and the absence of subgroup analyses limit the depth of our findings. It remains unclear whether the results apply equally to all professional groups within the emergency services (e.g., paramedics vs. firefighters). Finally, the relatively small and geographically specific sample restricts the generalizability of the findings. Since our data are rooted in the Swiss cultural context and healthcare system, the results may not be directly applicable to broader international populations or countries with different psychosocial support structures for rescue workers. Although the analysis controlled for pre-existing trauma, we cannot rule out that some PTSS stem from work-unrelated events. Furthermore, the lack of detailed information on the nature of the trauma (e.g., direct vs. witnessed) limits our ability to analyze differential triggers in this symptomatic pathway. Furthermore, resilience represents a crucial protective factor in managing traumatic stress. Individuals with higher resilient resources are less likely to develop severe PTSS and depressive symptoms and report lower rates of suicidal ideation. Since resilience was not assessed in the present study, it remains unclear to what extent varying resilience profiles may have influenced the observed associations.

Additionally, the model’s statistical parameters must be interpreted with caution. A *post-hoc* power analysis confirmed the study was severely underpowered to detect a moderate direct effect of trauma on suicidal ideation, suggesting the non-significant direct path may represent a Type II error rather than a true absence of effect. Furthermore, the exceptionally high R2 (69.4%) is largely an artifact of the matched case-control design; by establishing an artificial 50/50 prevalence of suicidal ideation, group-level variance was maximized. Consequently, this R2 does not reflect the model’s true predictive power for the general rescue worker population.

Notwithstanding these limitations, the present study offers a novel, theoretically grounded framework for understanding the progression from occupational trauma to suicidality. These findings underscore the need for prospective longitudinal studies using validated clinical interviews to corroborate the proposed symptomatic pathway and further elucidate the temporal dynamics of these relationships.

Crucially, the current data supports a Trauma-PTSS-Depression-Suicidal Ideation model. This indicates that suicide prevention in this group should not just focus on “suicide” directly, but rather on the proactive management of posttraumatic stress and depressive symptoms resulting from work-related trauma.

## Data Availability

The datasets presented in this study can be found in online repositories. The names of the repository/repositories and accession number(s) can be found below: Analysis pipeline to fully reproduce the results, tables, and figures of this paper are available at osf.io/c7xj2. A subset (replication dataset) of the raw data to reproduce the main results of this paper will be online during or after publication.
