# Supplementary material for: From work-related trauma to suicidal ideation: a serial mediation model of posttraumatic stress and depression in rescue workers
Source: Front Psychiatry. 2026 Apr 10;17:1805837. doi: 10.3389/fpsyt.2026.1805837 (PMC13106555; doi:10.3389/fpsyt.2026.1805837)
Supplement: Supplementary file 1 [file DataSheet1.pdf]

Supplementary Material

Table S1. Bivariate correlations among the four primary study variables: Trauma Exposure, Posttraumatic Stress Symptoms, Depressive Symptoms and Suicidal Ideation

	Trauma Exposure	Posttraumatic Stress Symptoms	Depressive Symptoms	Suicidal Ideation
Trauma Exposure	1			
Posttraumatic Stress Symptoms	0.349***	1		
Depressive Symptoms	0.166	0.708***	1	
Suicidal Ideation	0.246*	0.522***	0.604***	1

Note: r values are reported. Significance levels: * p < 0.05; ** p < 0.01; *** p < 0.001.

Saturated Serial Mediation Model Specification

To empirically justify the model constraints applied in the main manuscript, an initial saturated serial mediation model was estimated. This model specifies Trauma Exposure as the independent variable (*X*), PTSS as the first mediator (M_1), Depressive Symptoms as the second mediator (M_2), and Suicidal Ideation as the dichotomous dependent variable (*Y*). As a saturated model ($df = 0$), all possible direct and indirect paths between the variables were estimated, allowing for the inspection of the direct effect of Trauma on Depressive Symptoms and Suicidal Ideation alongside the mediated pathways. This step served to verify the full structure of associations and ensure no meaningful effects were overlooked. Non-significant direct paths identified in this saturated analysis were subsequently constrained to zero to improve model efficiency in the main manuscript.

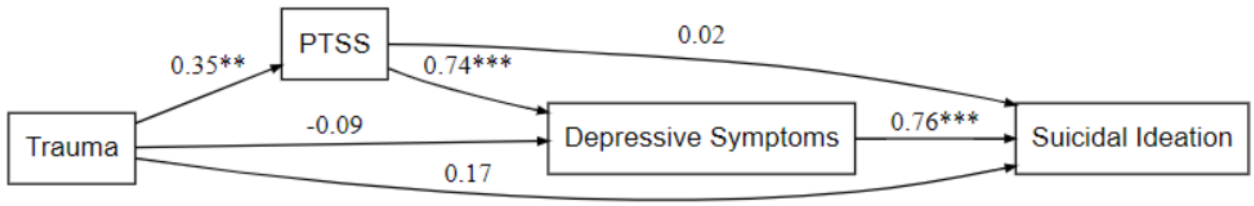

Figure S1. Path diagram illustrating the fully saturated model linking Trauma Load, Posttraumatic Stress Symptoms (PTSS), Depressive Symptoms, and Suicidal Ideation. Rectangles represent observed variables. Single-headed arrows represent regression paths. Values on the arrows indicate standardized path coefficients. The model accounts for all direct and indirect effects, including the serial pathway.

Significance levels: * p < 0.05; ** p < 0.01; *** p < 0.001.
